# Digital image analysis of ossification centers in the axial dens and body in the human fetus

**DOI:** 10.1007/s00276-016-1679-9

**Published:** 2016-04-29

**Authors:** Mariusz Baumgart, Marcin Wiśniewski, Magdalena Grzonkowska, Bogdan Małkowski, Mateusz Badura, Maria Dąbrowska, Michał Szpinda

**Affiliations:** 1 Department of Normal Anatomy, The Ludwik Rydygier Collegium Medicum in Bydgoszcz, The Nicolaus Copernicus University in Toruń, Łukasiewicza 1 Street, 85-821 Bydgoszcz, Poland; 2 Department of Positron Emission Tomography and Molecular Imaging, The Ludwik Rydygier Collegium Medicum in Bydgoszcz, The Nicolaus Copernicus University in Toruń, Łukasiewicza 1 Street, 85-821 Bydgoszcz, Poland

**Keywords:** Axis vertebra, Odontoid process, Ossification center, Size, Growth dynamics, Human fetus

## Abstract

**Purposes:**

The detailed understanding of the anatomy and timing of ossification centers is indispensable in both determining the fetal stage and maturity and for detecting congenital disorders. This study was performed to quantitatively examine the odontoid and body ossification centers in the axis with respect to their linear, planar and volumetric parameters.

**Methods:**

Using the methods of CT, digital image analysis and statistics, the size of the odontoid and body ossification centers in the axis in 55 spontaneously aborted human fetuses aged 17–30 weeks was studied.

**Results:**

With no sex difference, the best fit growth dynamics for odontoid and body ossification centers of the axis were, respectively, as follows: for transverse diameter *y* = −10.752 + 4.276 × ln(age) ± 0.335 and *y* = −10.578 + 4.265 × ln(age) ± 0.338, for sagittal diameter *y* = −4.329 + 2.010 × ln(age) ± 0.182 and *y* = −3.934 + 1.930 × ln(age) ± 0.182, for cross-sectional area *y* = −7.102 + 0.520 × age ± 0.724 and *y* = −7.002 + 0.521 × age ± 0.726, and for volume *y* = −37.021 + 14.014 × ln(age) ± 1.091 and *y* = −37.425 + 14.197 × ln(age) ± 1.109.

**Conclusions:**

With no sex differences, the odontoid and body ossification centers of the axis grow logarithmically in transverse and sagittal diameters, and in volume, while proportionately in cross-sectional area. Our specific-age reference data for the odontoid and body ossification centers of the axis may be relevant for determining the fetal stage and maturity and for in utero three-dimensional sonographic detecting segmentation anomalies of the axis.

## Introduction

The detailed understanding of the morphology and timing of ossification centers is indispensable in the prenatal assessment, particularly in both determining the fetal stage and maturity and for detecting segmentation anomalies of the fetal spine [12, 21, 25, 27, 28]. The process of spine ossification had primarily been elucidated due to histological and radiographic methods, and as modern diagnostic methods were developing, two- and three-dimensional ultrasound and computed tomography were consecutively engaged [7, 12, 26]. With the exception of coccygeal vertebrae, there are three ossification centers per a vertebra: one in its body and one in either neural arch [1–3, 12, 21]. The first vertebral body ossification centers occur in the arches of upper cervical vertebrae, as early as at week 8 in the axis, and then progress caudad [6]. The first vertebral body ossification centers occur at week 10 in lower thoracic vertebrae and the first lumbar vertebra, from which the process continues both cephalad and caudad [20].

Reports in the professional literature unveiled substantial differences in the development of the unique cervical vertebrae, i.e., atlas and axis. In the atlas, three ossification centers were found: one located within the anterior arch and two located within the posterior arch. However, four ossification centers were found in the axis: one in the body, one in the dens and one in either neural arch [7, 13, 15, 17]. Because of flexion–extension at the atlantooccipital joint and rotation at the atlantoaxial joint, 3-dimensional sonographic evaluation of the atlas and axis in utero fetuses may frequently misjudge their malalignment or offset phenomena [12]. To date, the quantitative analysis of some linear, planar and spatial dimensions of ossification centers of the spine has been established in detail using computed tomography and digital image analysis only for the C4 [5], T6 [23] and L3 [24] vertebrae. Therefore, this study involved advanced morphometric analysis of the non-typical axial vertebra, the dens of which from a developmental point of view is actually the atlantal body.

Taking into account all of the above, the purposes of the present study were to accomplish:

- morphometric analysis of the ossification centers in the axial dens and body with respect to their linear, planar and volumetric parameters as presumptive age-specific reference data,
- possible differences between sexes for the parameters studied, and
- growth dynamics for the analyzed parameters, expressed by best fit mathematical models.

## Materials and methods

**Fig. 1:**
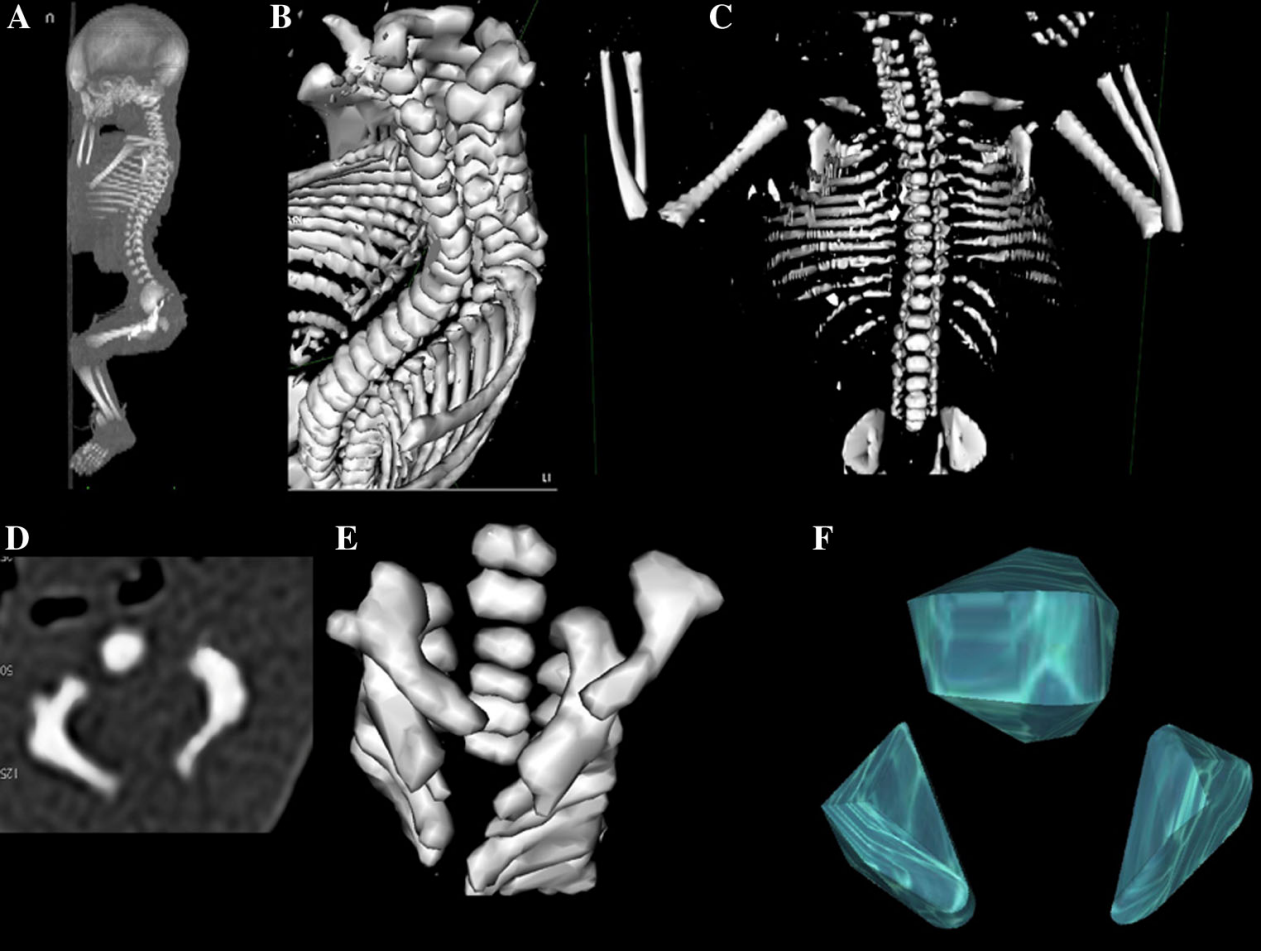
CT of a female fetus aged 23 weeks (**a**) recorded in DICOM formats and assessed by Osirix 3.9 in horizontal (**b**) and frontal (**c**) planes, with the transverse view of cervical vertebrae (**d**), reconstruction of the atlas and axis (**e**), and the body and neural arches of axis (**f**)

**Fig. 2:**
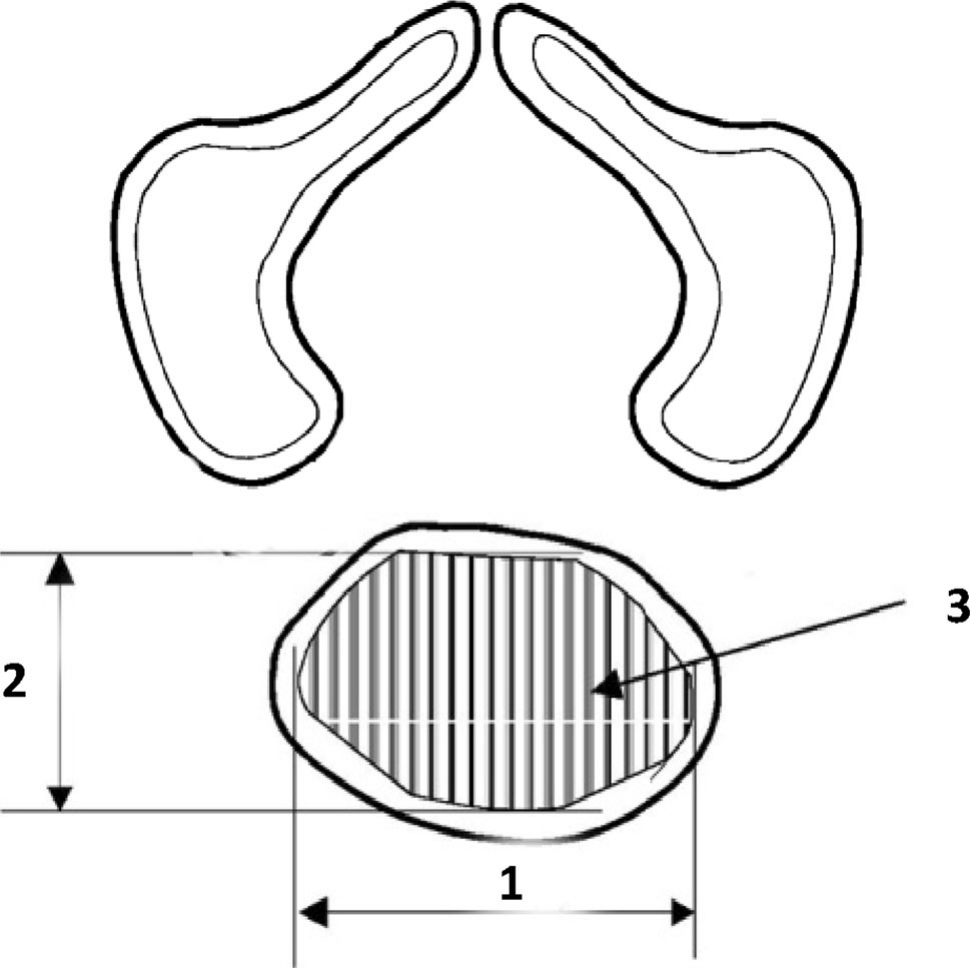
Diagram showing obtained measurements of the odontoid and body ossification centers of the axis

The study material encompassed 55 human fetuses of both sexes (27 males and 28 females) aged 17–30 weeks, derived from spontaneous abortions and preterm deliveries. The material was acquired before the year 2000 and has belonged to the specimen collection of our Department of Normal Anatomy. The experiment was sanctioned by the Bioethics Committee of the University (KB 275/2011). The fetal age was based on the crown-rump length. Table 1 lists the characteristics of the study group, including age, number and sex of the fetuses. Using the Siemens-Biograph 128 mCT camera, the fetuses were scanned at a step of 0.4 mm, and recorded in DICOM formats (Fig. 1). For each individual, a total of 10 linear, planar and volumetric measurements of the axis were completed (Fig. 2). Although the axial dens and body were still cartilaginous, their outlines were already clearly visible, and so facilitating their volumetric assessment [8, 10].

**Table 1:** Age, number and sex of the fetuses studied

Gestational age	Crown-rump length (mm)				Number of fetuses	Sex	
Weeks (Hbd-life)	Mean	SD	Min.	Max.		♂	♀
17	115.00		115.00	115.00	1	0	1
18	133.33	5.77	130.00	140.00	3	1	2
19	149.50	3.82	143.00	154.00	8	3	5
20	161.00	2.71	159.00	165.00	4	2	2
21	174.75	2.87	171.00	178.00	4	3	1
22	185.00	1.41	183.00	186.00	4	1	3
23	197.60	2.61	195.00	202.00	5	2	3
24	208.67	3.81	204.00	213.00	9	5	4
25	214.00		214.00	214.00	1	0	1
26	229.00	5.66	225.00	233.00	2	1	1
27	237.50	3.33	233.00	241.00	6	6	0
28	249.50	0.71	249.00	250.00	2	0	2
29	253.00	0.00	253.00	253.00	2	0	2
30	263.25	1.26	262.00	265.00	4	3	1
Total					55	27	28

**Fig. 3:**
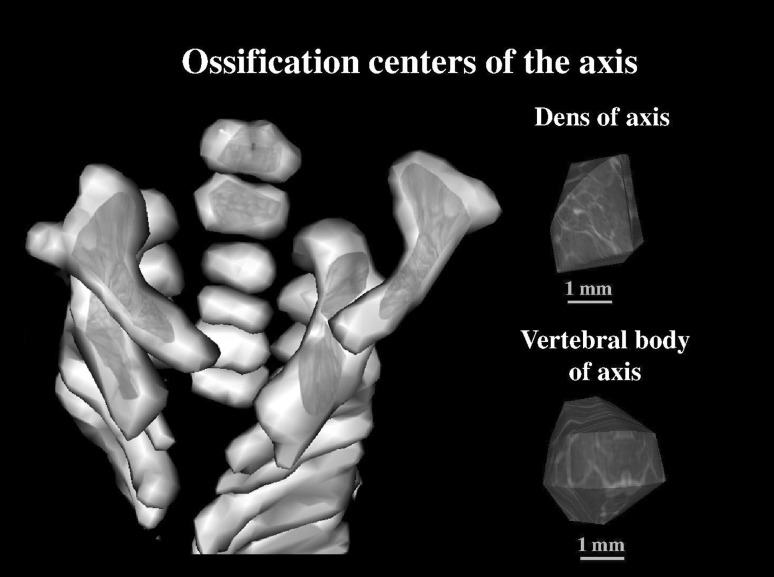
Bone reconstruction of cervical vertebrae and the odontoid and body ossification centers of the axis

The following 10 parameters of ossification centers within the axial dens and body were measured:

- 1, 2 transverse diameter, expressed by the maximal distance between the right and left borderlines of the ossification center in the transverse plane (Fig. 2),
- 3, 4 sagittal diameter, expressed by the maximal distance between the anterior and posterior borderlines of the ossification center in the sagittal plane (Fig. 2),
- 5, 6 cross-sectional area, based on the determined contour of the ossification center in the transverse plane (Fig. 2), and
- 7, 8 volume of the axial dens and body ossification centers, respectively, calculated using advanced diagnostic imaging tools for 3D reconstruction, taking into account both the position and absorption of radiation by bone tissue (Fig. 3).

Furthermore, the two volumetric calculations of the axial dens (9) and body (10) were involved.

With respect to the odontoid or body ossification centers of the axis, their sagittal-to-transverse ratios were calculated as the quotient of their sagittal and transverse diameters. The odontoid or body ossification centers volume ratios were offered to determine proportions between the volumes of the axial odontoid and body ossification centers and the volumes of the axial dens and body, respectively.

The algebraic data were subjected to statistical analysis. Distribution of variables was checked using the Shapiro–Wilk test, while homogeneity of variance was checked using Fisher’s test. Due to normality of distribution, the results have been expressed as arithmetic means with standard deviation (SD). To compare the means, Student’s *t* test for independent variables and one-way analysis of variance were used, followed by post hoc Tukey’s comparisons. With no similarity of variance, the non-parametric Kruskal–Wallis test was used. So as to examine sex differences, firstly we tested possible differences between the following five age groups: 17–19, 20–22, 23–25, 26–28, and 29–30 weeks of gestation. Secondly, we checked sex differences for the whole examined group, without taking into account fetal ages. The growth dynamics for the analyzed parameters were based on linear and nonlinear regression analysis. The match between the modelled functions and numerical data was evaluated on the base of the coefficient of determination (*R* ^2^).

## Results

The numerical results for all analyzed parameters of the odontoid and body ossification centers in the axis in the human fetus aged 17–30 weeks have been displayed in Tables 2, 3 and 4.

**Table 2:** Transverse and sagittal diameters of the odontoid and body ossification centers of the axis

Gestational age (weeks)	Number of fetuses	Odontoid ossification center of axis				Body ossification center of axis			
		Transverse diameter (mm)		Sagittal diameter (mm)		Transverse diameter (mm)		Sagittal diameter (mm)	
		Mean	SD	Mean	SD	Mean	SD	Mean	SD
17	1	1.43		1.34		1.74		1.47	
18	3	1.54	0.15	1.40	0.15	1.73	0.21	1.56	0.15
19	8	1.45	0.12	1.46	0.12	1.57	0.11	1.63	0.12
		↓(*P* < 0.01)		↓(*P* < 0.01)		↓(*P* < 0.01)		↓(*P* < 0.01)	
20	4	2.19	0.22	1.65	0.18	2.30	0.25	1.83	0.22
21	4	2.67	0.12	2.06	0.06	2.79	0.11	2.16	0.03
22	4	2.71	0.38	1.88	0.25	2.83	0.42	2.03	0.23
		↓(*P* < 0.01)		↓(*P* < 0.01)		↓(*P* < 0.01)		↓(*P* < 0.01)	
23	5	2.41	0.35	1.90	0.12	2.57	0.32	2.01	0.19
24	9	2.94	0.23	2.16	0.11	3.07	0.22	2.32	0.08
25	1	2.96		2.06		3.08		2.23	
		↓(*P* < 0.001)		↓(*P* < 0.001)		↓(*P* < 0.001)		↓(*P* < 0.001)	
26	2	3.26	0.26	2.14	0.13	3.45	0.17	2.35	0.07
27	6	3.21	0.34	2.26	0.18	3.36	0.33	2.40	0.14
28	2	3.47	0.25	2.20	0.04	3.62	0.24	2.26	0.09
		↓(*P* < 0.01)		↓(*P* < 0.01)		↓(*P* < 0.01)		↓(*P* < 0.01)	
29	2	3.32	0.00	2.54	0.00	3.48	0.01	2.71	0.02
30	4	3.69	0.28	2.40	0.21	3.84	0.32	2.48	0.20

**Table 3:** Cross-sectional area and volume of the odontoid and body ossification centers the of axis

Gestational age (weeks)	Number of fetuses	Odontoid ossification center of axis				Body ossification center of axis			
		Cross-sectional area (mm^2^)		Volume (mm^3^)		Cross-sectional area (mm^2^)		Volume (mm^3^)	
		Mean	SD	Mean	SD	Mean	SD	Mean	SD
17	1	1.65		2.84		1.80		2.91	
18	3	2.09	0.22	3.48	0.30	2.23	0.21	3.67	0.33
19	8	2.30	0.28	3.43	0.18	2.43	0.27	3.57	0.21
		↓(*P* < 0.001)		↓(*P* < 0.01)		↓(*P* < 0.001)		↓(*P* < 0.01)	
20	4	3.49	0.29	4.72	0.47	3.60	0.29	4.89	0.45
21	4	4.36	0.15	6.94	0.14	4.48	0.17	7.10	0.10
22	4	4.59	1.05	6.17	1.58	4.70	1.04	6.29	1.58
		↓(*P* < 0.01)		↓(*P* < 0.001)		↓(*P* < 0.01)		↓(*P* < 0.001)	
23	5	4.28	0.91	6.14	1.48	4.44	0.94	6.28	1.48
24	9	5.50	0.70	7.75	0.97	5.62	0.73	7.91	0.97
25	1	5.62		8.68		5.80		8.96	
		↓(*P* < 0.001)		↓(*P* < 0.001)		↓(*P* < 0.001)		↓(*P* < 0.001)	
26	2	6.36	0.94	8.90	0.07	6.50	0.99	9.04	0.19
27	6	6.74	1.28	9.18	1.24	6.86	1.27	9.36	1.29
28	2	6.62	0.93	9.45	1.02	6.70	0.85	9.68	1.16
		↓(*P* < 0.001)		↓(*P* < 0.001)		↓(*P* < 0.001)		↓(*P* < 0.001)	
29	2	7.39	0.09	9.64	0.01	7.55	0.07	9.74	0.02
30	4	8.55	0.28	10.08	1.42	8.72	0.27	10.39	1.57

**Table 4:** Volume of the axial dens and body

Gestational age (weeks)	Number of fetuses	Volume of axial dens (mm^3^)		Volume of axial body (mm^3^)	
		Mean	SD	Mean	SD
17	1	11.50		12.05	
18	3	16.18	0.42	17.24	0.31
19	8	19.03	5.74	20.42	6.07
		↓(*P* < 0.001)		↓(*P* < 0.001)	
20	4	19.03	1.24	20.42	1.74
21	4	31.06	2.00	33.47	1.85
22	4	23.60	4.85	24.82	5.29
		↓(*P* < 0.01)		↓(*P* < 0.01)	
23	5	26.11	5.45	27.92	5.61
24	9	32.33	5.98	34.04	5.93
25	1	31.39		34.03	
		↓(*P* < 0.01)		↓(*P* < 0.01)	
26	2	43.85	2.10	45.68	2.64
27	6	44.17	4.57	47.20	6.07
28	2	56.60	2.51	59.52	2.58
		↓(*P* < 0.01)		↓(*P* < 0.01)	
29	2	39.67	0.42	41.18	0.33
30	4	55.96	6.26	54.73	8.92

**Fig. 4:**
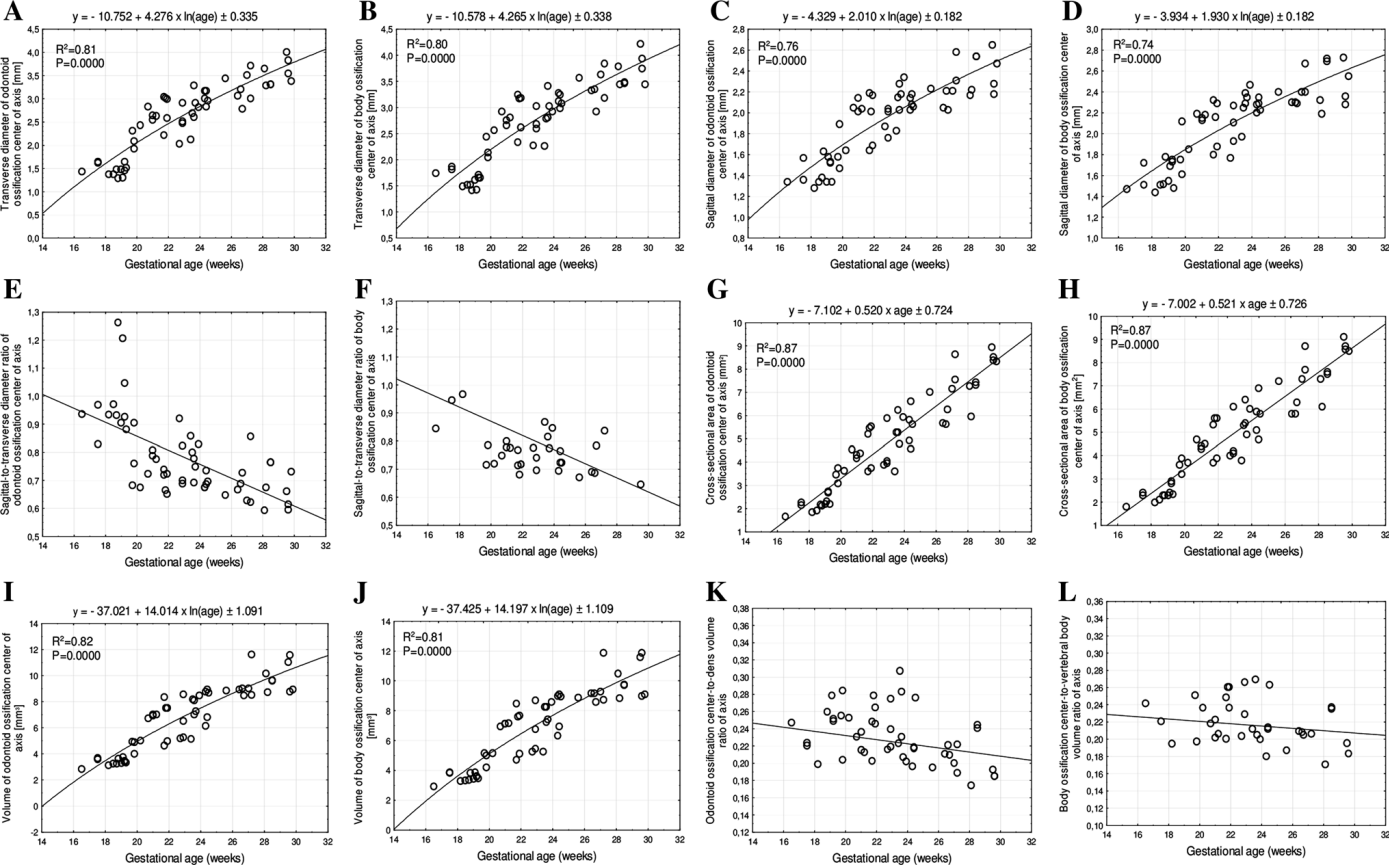
Regression lines for transverse diameter of the odontoid (**a**) and body (**b**) ossification centers, for sagittal diameter of the odontoid (**c**) and body (**d**) ossification centers, for sagittal-to-transverse diameter ratio of the odontoid (**e**) and body (**f**) ossification centers, for cross-sectional area of the odontoid (**g**) and body (**h**) ossification centers, for volume of the odontoid (**i**) and body (**j**) ossification centers, and for the odontoid (**k**) and body (**l**) ossification centers volume ratios of the axis

The mean transverse diameters of the odontoid and axial body ossification centers ranged from 1.43 to 3.69 mm and from 1.74 to 3.84 mm, respectively. The transverse diameters of these two ossification centers increased logarithmically as follows: *y* = −10.752 + 4.276 × ln(age) ± 0.335 (*R* ^2^ = 0.81) in the dens (Fig. 4a), and *y* = −10.578 + 4.265 × ln(age) ± 0.338 (*R* ^2^ = 0.80) in the body of the axis (Fig. 4b).

The mean sagittal diameters of the odontoid and axial body ossification centers increased from 1.34 to 2.40 mm and from 1.47 to 2.48 mm, correspondingly. The odontoid and body ossification centers grew in sagittal diameter in accordance with the following logarithmic fashions: *y* = −4.329 + 2.010 × ln(age) ± 0.182 (*R* ^2^ = 0.76) and *y* = −3.934 + 1.930 × ln(age) ± 0.182 (*R* ^2^ = 0.74), respectively (Fig. 4c, d).

In the study period, the mean value of the sagittal-to-transverse diameter ratio decreased from 0.91 to 0.68 (Fig. 4e) and from 0.88 to 0.68 (Fig. 4f) for the odontoid and body ossification centers of the axis, respectively.

The mean cross-sectional area of the axial ossification centers increased from 1.65 to 8.55 mm^2^ in the dens and from 1.80 to 8.72 mm^2^ in the body of the axis, and modelled the linear functions *y* = −7.102 + 0.520 × age ± 0.724 (*R* ^2^ = 0.87) and *y* = −7.002 + 0.521 × age ± 0.726 (*R* ^2^ = 0.87), respectively (Fig. 4g, h).

The mean volume of the odontoid and body ossification centers crept up from 2.84 to 10.08 mm^3^ and from 2.91 to 10.39 mm^3^, respectively. This corresponded to the logarithmic models, expressed by: *y* = −37.021 + 14.014 × ln(age) ± 1.091 (*R* ^2^ = 0.82) for the odontoid ossification center (Fig. 4i) and *y* = −37.425 + 14.197 × ln(age) ± 1.109 (*R* ^2^ = 0.81) for the body ossification center (Fig. 4j) of the axis.

During the analyzed period, the dens and axial body revealed a respective increase in volume from 11.5 to 55.96 mm^3^ and from 12.05 to 54.73 mm^3^, but the odontoid and body ossification centers volume ratios decreased from 0.22 to 0.19 (Fig. 4k) and from 0.21 to 0.20, correspondingly (Fig. 4l).

## Discussion

Reports in the professional literature present divergent data on the existence of ossification centers in vertebral bodies and arches. Bagnall et al. [1–3] observed that ossification centers in vertebral bodies initially appeared in the inferior thoracic–superior lumbar spine, i.e., vertebrae T11, T12 and L1. The further ossification process concurrently progressed both cephalad and caudad. On the other hand, the ossification of vertebral arches simultaneously started in the cervical, lower thoracic and upper lumbar segments. According to these authors, the commencement of ossification in neural arches might be both a consequence of fetal movements and the influence of particular skeletal muscles. Skórzewska et al. [20] found vertebrae to start to ossify in fetuses aged 10–11 weeks. Firstly, ossification centers appeared within the neural arches of the cervical and upper thoracic vertebrae, and 1 week later were also present in the arches of the successive thoracic and lumbar vertebrae. The presence of body ossification centers was reported in the 10-week fetus within vertebrae T6–L1. This confirmed that vertebral arch ossification progressed caudad, while vertebral body ossification followed both cephalad and caudad directions.

Both the atlas and odontoid process (dens) are derivatives of the first cervical sclerotome, whereas the remainder of the axis develops from the second cervical sclerotome [21]. Thus, from a strict point of view the odontoid process presents an atlantal body [4]. It is commonly stated that the axis possesses four primary ossification centers, solitary located in its body, dens and left and right neural processes. Four ossification centers of the axis were visualized in both fetuses [7] and children [16]. On the contrary, Piatt and Grissom [19] showed that children had five ossification centers, since two of them occurred in the central and apical parts of the dens. Therefore, the dens is formed from two separate primary ossification centers that fuse at week 28 of gestation, while the secondary ossification center appears in its apical part between 3 and 6 years, and fuses as late as around 12 years. The axial body fuses with the dens between 3 and 6 years, but the fusion site is conspicuous until the age of 11 years and may imitate dens fracture. In turn, the axial neural processes fuse around the age of 2–3 years [17, 19]. Vignolo et al. [26] reported that ossification started earlier in female fetuses, thus male fetuses were more difficult to assess in this respect. The present paper and our previous findings [4, 5, 22–24] have not confirmed any sex differences in ossification of the spine. Therefore, in the present study both numerical data and statistical analysis are presented aggregately without taking sex into account.

Castellana and Kosa [7] showed that it was possible to precisely estimate the age and body length of the fetus solely based on the size of the axial and atlantal ossification centers. According to these authors, numerical data specifying the developing axial dens may be useful in determining fetal age. Kosa and Castellana [14] found the size of the vertebral ossification centers to increase proportionately with the exception of the odontoid ossification center, which displayed allometric growth. Szpinda et al. [22] presented a cross-sectional study of the ossification center of the C1–S5 vertebral bodies in 55 human fetuses aged 17–30. Its transverse diameter gradually increased from the C1 to T12 vertebra, stabilized through vertebrae L1–L3, and decreased from the L4 to S5 vertebra. Its sagittal diameter increased from the C1 to T5 vertebra, stabilized for vertebrae T6–T9, decreased for vertebrae T10–T12, increased for vertebrae L1 and L2, and finally decreased for vertebrae L3–S5. Its cross-sectional area gradually increased from the C1 to L2 vertebra, and decreased from the L3 to S5 vertebra. Its volume gradually increased from the C1 to L3 vertebra, and sharply decreased from the L4 to S5 vertebra. Of note, on the same fetal material Szpinda and his collaborators carried out a comprehensive morphometric analysis including growth curves for typical vertebrae C4 [5], T6 [23] and L3 [24]. However, Castellana and Kosa [7] were the only authors to measure the axial ossification centers in a large sample, consisting of 106 human fetuses aged 16–40 weeks. Therefore, the numerical findings obtained by Szpinda’s team [4, 23, 24] and Castellana and Kosa [7] are indispensable to complete this discussion. In the material under examination, both transverse and sagittal diameters of the odontoid and body ossification centers of the axis grew in accordance with a natural logarithmic regression. In the axial dens and body, the transverse diameters of ossification centers modelled the following logarithmic functions: *y* = −10.752 + 4.276 × ln(age) and *y* = −10.578 + 4.265 × ln(age), respectively. The sagittal diameters of the odontoid and body ossification centers increased logarithmically as follows: *y* = −4.329 + 2.010 × ln(age) and *y* = −3.934 + 1.930 × ln(age), respectively. Surprisingly enough, according to Castellana and Kosa [7], the odontoid ossification center grew as follows: *y* = 2.652 (dens width) + 1.110 (dens height) + 1.392 (dens depth) + 16.932. Furthermore, the axial body ossification center followed the formula: *y* = 4.063 (body width) + 2.857 (body depth) − 0.469 (body height) + 10.530. As reported in the medical literature, the vertebral body ossification centers revealed a natural logarithmic increase in both transverse and sagittal diameters. This was substantiated by Baumgart et al. [5] in relation to vertebra C4 (*y* = −8.836 + 3.708 × ln(age) and *y* = −7.748 + 3.240 × ln(age), respectively) and by Szpinda et al. [23, 24] in relation to both vertebrae T6 (*y* = −14.784 + 6.115 × ln(age) and *y* = −12.065 + 5.019 × ln(age), respectively) and L3 (*y* = −27.610 + 10.341 × ln(age) and *y* = −13.858 + 5.636 × ln(age), respectively).

In the material under examination, noteworthy was the intense growth of the axial ossification centers in their transverse diameters when compared to their sagittal diameters. This was confirmed by the sagittal-to-transverse diameter ratio that decreased from 0.91 to 0.68 for the odontoid ossification center and from 0.88 to 0.68 for the body ossification center of the axis. An analogous finding referred to the body ossification center of vertebra L3, in which the sagittal-to-transverse diameter ratio declined from 1.05 to 0.62. Contrary to the axial ossification centers, a more intense increase in sagittal diameter was observed in vertebrae C4—from 0.86 to 0.88 and T6—from 0.81 to 0.85.

The odontoid and body ossification centers of the axis increased in cross-sectional area in a commensurate fashion: *y* = −7.102 + 0.520 × age ± 0.724 and *y* = −7.002 + 0.521 × age ± 0.726, respectively. Of note, such a proportionate increase in cross-sectional area was previously proved by Baumgart et al. [5] for vertebra C4 as *y* = −4.690 + 0.437 × age, and by Szpinda et al. [23, 24] for both vertebrae T6: *y* = −15.591 + 1.200 × age and L3: *y* = −32.423 + 2.071 × age.

We demonstrated that the volumetric growth both the odontoid and body ossification centers of the axis in relation to fetal age generated the consecutive logarithmic functions: *y* = −37.021 + 14.014 × ln(age) and *y* = −37.425 + 14.197 × ln(age), respectively. Typical of these functions is a gradually decreasing volumetric growth rate with fetal age. Thus, such a logarithmic volumetric growth in the material under examination is rather unanticipated because it does not correspond with a proportionate increase in ossification center volume concerning vertebral bodies of typical vertebrae: C4 as *y* = −5.917 + 0.582 × age [5], T6 as *y* = −22.120 + 1.663 × age [23], and L3 as *y* = −44.200 + 2.823 × age [24].

Between weeks 17 and 30 of gestation, the mean volume of the axial dens and body volume raised from 11.5 to 55.96 mm^3^, and from 12.05 to 54.73 mm^3^, respectively. The mean volumetric growth of vertebral bodies was found to gain from 15.53 to 72.43 mm^3^ for vertebra C4 [5], from 32.54 to 158.14 mm^3^ for vertebra T6 [23], and from 14.50 to 41.65 mm^3^ for vertebra L3 [24]. In the material under examination, both the odontoid and body ossification centers volume ratios were decreasing with age from 0.22 to 0.19 and from 0.21 to 0.20, respectively. A similar phenomenon was observed with relation to body ossification centers of other vertebrae: from 0.23 to 0.21 for C4 [5], from 0.28 to 0.21 for T6 [23], and from 0.24 to 0.14 for L3 [24].

The uniqueness of our study results in both numerical data and computed nomograms for the growing ossification centers of the axis in the human fetus. This may noticeably hone our quantitative morphology concerning advances in ossification of the fetal axis, thereby enabling to determine the size of odontoid and body ossification parameters of the axis in accordance with gestational age. This may be germane when monitoring normal fetal growth and screening for inherited faults in fetuses suffering from segmental anomalies of the spine. It should be emphasized that the odontoid and vertebral body ossification centers of the axis can be visualized, and so subjected to 3-dimensional sonography as early as at 13 weeks of gestation [12]. Of note, the first and second cervical vertebrae in the fetus are problematic to discern their normal from abnormal development. According to Henderson et al. [12], as a result of the rotational capability at the atlantoaxial joint, actually normal formation of the cervical spine may be misapprehended as unusual segmental anomalies. These suspected malalignments of vertebrae C1 and C2 mostly refer to some parasagittal and coronal planes. As reported, due to head rotation from 2° to 36°, in 2/3 of the fetuses studied, the odontoid ossification center did not entirely align with the axial body ossification center, and so the lateral offset vacillated from 0.0 to 3.3 mm. The produced offset could imitate a segmentation anomaly of the cervical spine. To its specific categories belong both hemivertebrae and butterfly vertebrae [12]. Hemivertebra is a consequence of unilateral aplasia of either right or left chondrification centers in the vertebral body that normally should have fused into one vertebral body ossification center. As a result, a triangular cuneiform vertebral body is responsible for considerable sagittal and coronal malalignments of the spine. Evidently, when compared to normal vertebrae, the respective hemivertebrae in the fetus must be characterized by a much smaller size of osseous structures, i.e. body ossification centers. We speculate that hemivertebral body ossification centers, including odontoid and body ossification centers of the axis, may be reduced by roughly 50 % in their transverse diameter, cross-sectional area and volume when compared to our age-specific reference data obtained in the material under examination. As far as butterfly vertebra is concerned, it displays two halves of the vertebral body that failed to coalesce because of the persistent notochord that disconnected them. When compared to our age-specific reference data, the size of body ossification centers of butterfly vertebrae, including those of the axial body and dens, may reveal a substantial decrease in sagittal diameter, cross-sectional area and volume. As stated by Henderson et al. [12], the whole fetal spine should be assessed because one segmentation anomaly is often accompanied by the second one. We believe that our numerical data achieved in the present study may be conducive when dealing with some anomalies typical of the axis, i.e., the os odontoideum, condylus tertius, ossiculum terminale, and odontoid agenesis. The os odontoideum (odontoid bone) is characterized by the properly developed dens, which failed to fuse onto the axial body, so exists as a separate bone. In the condition known as the condylus tertius (third condyle), the axial dens presents a small individual bone, which is linked in either ligamentous or direct ways with the margin of the foramen magnum or anterior atlantal arch. In these cases it may deceptively imitate one more condyle, that is the third occipital condyle. The ossiculum terminale (terminal ossicle) is characterized by a separate apical part, not united with the remaining part of the dens. Odontoid agenesis results in the absence of the odontoid basilar or apical segments, or the whole dens. Both odontoid agenesis and the non-ossified odontoid process may destabilize the middle atlanto-occipital joint, leading to its subluxation. This defect may accompany synostosis of vertebrae C2 and C3, axial body malformation, and occipital vertebrae [9, 11, 18].

## Conclusions

1. With no sex differences, the odontoid and body ossification centers of the axis grow logarithmically in transverse and sagittal diameters, and in volume, while proportionately in cross-sectional area.
2. Our specific-age reference data for the odontoid and body ossification centers of the axis may be relevant for determining the fetal stage and maturity and for in utero three-dimensional sonographic detecting segmentation anomalies of the axis.

## References

[1] Bagnall KM, Harris PF, Jones PR (1977). A radiographic study of the human fetal spine. 1. The development of the secondary cervical curvature. J Anat.

[2] Bagnall KM, Harris PF, Jones PR (1977). A radiographic study of the human fetal spine 2. The sequence of development of ossification centres in the vertebral column. J Anat.

[3] Bagnall KM, Harris PF, Jones PR (1979). A radiographic study of the human fetal spine 3. Longitudinal growth. J Anat.

[4] Baumgart M (2012) Morphometric study of the vertebral column in human fetuses. Doctoral thesis, Bydgoszcz, pp 1–208 (in Polish)

[5] Baumgart M, Szpinda M, Szpinda A (2013). New anatomical data on the growing C4 vertebra and its three ossification centers in human fetuses. Surg Radiol Anat.

[6] Castellana C, Kósa F (1999). Morphology of the cervical vertebrae in the fetal-neonatal human skeleton. J Anat.

[7] Castellana C, Kósa F (2001). Estimation of fetal age from dimensions of atlas and axis ossification ceneters. Forensic Sci Int.

[8] Chano T, Matsumoto K, Ishizawa M, Morimoto S, Hukuda S, Okabe H, Kato H, Fujino S (1996). Analysis of the presence of osteocalcin, S-100 protein, and proliferating cell nuclear antigen in cells of various types of osteosarcomas. Eur J Histochem.

[9] Currarino G, Rollins N, Diehl JT (1994). Congenital defects of the posterior arch of the atlas: a report of seven cases including an affected mother and son. Am J Neuroradiol.

[10] Duarte WR, Shibata T, Takenaga K, Takahashi E, Kubota K, Ohya K, Ishikawa I, Yamauchi M, Kasugai S (2003). S100A4: a novel negative regulator of mineralization and osteoblast differentiation. J Bone Miner Res.

[11] Hasan M, Shukla S, Siddiqui MS, Singh D (2001). Posterolateral tunnels and ponticuli in human atlas vertebrae. J Anat.

[12] Henderson P, Desai IP, Pettit K, Benke S, Brouha SS, Romine LE, Beeker K, Chuang NA, Yaszay B, Van Houten L, Pretorius DH (2016). Evaluation of fetal first and second cervical vertebrae: normal or abnormal?. J Ultrasound Med.

[13] Junewick JJ, Chin MS, Meesa IR, Ghori S, Boynton SJ, Luttenton CR (2011). Ossification patterns of the atlas vertebra. AJR Am J Roentgenol.

[14] Kósa F, Castellana C (2005). New forensic anthropological approachment for the age determination of human fetal skeletons on the base of morphometry of vertebral column. Forensic Sci Int.

[15] Lee HJ, Kim JT, Shin MH, Choi DY, Hong JT (2015). Quantification of pediatric cervical spine growth at the cranio-vertebral junction. J Korean Neurosurg Soc.

[16] Lee HJ, Kim JT, Shin MH, Choi DY, Park YS, Hong JT (2015). The ossification pattern in paediatric occipito-cervical spine: is it possible to estimate real age?. Clin Radiol.

[17] Lustrin ES, Karakas SP, Ortiz AO, Cinnamon J, Castillo M, Vaheesan K, Brown JH, Diamond AS, Black K, Singh S (2003). Pediatric cervical spine: normal anatomy, variants, and trauma. Radiographics.

[18] Martel W, Bole GG (1968). Pathologic fracture of the odontoid process in the rheumatoid arthritis. Radiology.

[19] Piatt JH, Grissom LE (2011). Developmental anatomy of the atlas and axis in childhood by computed tomography. J Neurosurg Pediatr.

[20] Skórzewska A, Grzymisławska M, Bruska M, Łupicka J, Woźniak W (2013). Ossification of the vertebral column in human foetuses: histological and computed tomography studies. Folia Morphol.

[21] Swischuk LE (2013) Developmental anatomy. In: Imaging of the cervical spine in children. Springer, New York, pp 1–9

[22] Szpinda M, Baumgart M, Szpinda A, Woźniak A, Małkowski B, Wiśniewski M, Mila-Kierzenkowska C, Króliczewski D (2013). Cross-sectional study of the ossification center of the C1–S5 vertebral bodies. Surg Radiol Anat.

[23] Szpinda M, Baumgart M, Szpinda A, Woźniak A, Mila-Kierzenkowska C, Dombek M, Kosiński A, Grzybiak M (2013). Morphometric study of the T6 vertebra and its three ossification centers in the human fetus. Surg Radiol Anat.

[24] Szpinda M, Baumgart M, Szpinda A, Woźniak A, Mila-Kierzenkowska C (2013). New patterns of the growing L3 vertebra and its 3 ossification centers in human fetuses—a CT, digital, and statistical study. Med Sci Monit Basic Res.

[25] Varras M, Akrivis C (2010). Prenatal diagnosis of fetal hemivertebra at 20 weeks’ gestation with literature review. Int J Gen Med.

[26] Vignolo M, Ginocchio G, Parodi A, Torrisi C, Pistorio A, Venturini PL, Aicardi G, De Biasto P (2005). Fetal spine ossification: the gender and individual differences illustrated by ultrasonography. Ultrasound Med Biol.

[27] Wax JR, Watson WJ, Miller RC, Ingardia CJ, Pinette MG, Cartin A, Grimes CK, Blackstone J (2008). Prenatal sonographic diagnosis of hemivertebrae: associations and outcomes. J Ultrasound Med.

[28] Wei Q, Cai A, Wang X, Xie L, Wang B, Wang X (2013). Value of 3-dimensional sonography for prenatal diagnosis of vertebral formation failure. J Ultrasound Med.

